# How Does Happiness Influence the Loyalty of Karate Athletes? A Model of Structural Equations From the Constructs: Consumer Satisfaction, Engagement, and Meaningful

**DOI:** 10.3389/fpsyg.2021.653034

**Published:** 2021-04-07

**Authors:** Estela Núñez-Barriopedro, Pedro Cuesta-Valiño, Pablo Gutiérrez-Rodríguez, Rafael Ravina-Ripoll

**Affiliations:** ^1^Economics and Business Management Department, Faculty of Economics, Business and Tourism, University of Alcalá, Alcalá de Henares, Spain; ^2^Department of Business Administration, Faculty of Economic and Management Sciences, University of León, León, Spain; ^3^Department of Business Management, Faculty of Economic and Business Sciences, INDESS, University of Cadiz, Cadiz, Spain

**Keywords:** happiness, loyalty, consumer satisfaction, commitment, quality, engagement, meaningful, SEM

## Abstract

Federations are concerned about attracting new sportsmen and sportswomen and increasing the number of members. The purpose of this research was to describe karate federations' strategies for attracting and retaining members through happiness. The analysis was carried out by designing a structural equation modeling (SEM), which allowed to analyze the main variables that influenced the happiness of the karate athlete and consequently to study their effect on people's loyalty to sports federations. In particular, Partial least squares SEM was applied in an overall model when it was possible to understand the happiness role in relation with other traditional relevant variables on loyalty. The data were obtained through primary sources employing a survey sent to the autonomous federations in the discipline of karate, obtaining a sample of 682 federated members in Spain. The results of the model revealed that consumer satisfaction, engagement, and meaningful influence on consumer happiness, but engagement was the most important and relevant variable for affecting this variable. Finally, consumer satisfaction and consumer happiness influence loyalty, and consumer satisfaction was the most important variable, but consumer happiness showed a real alternative for improving loyalty in karate sports federations. Then, one of the implications of this work was that it helped to explain how the federations can be managed to achieve loyal consumers together with a more considerable increase in the number of federated members.

## Introduction

Consumer loyalty is a strategic variable that ensures the survival of entities in the long term and has therefore been extensively researched in the literature from the point of view of psychology, marketing, and management (Dwyer et al., 1987; Dick and Basu, 1994; Sheth, 1996). It is generally accepted that consumer satisfaction has a positive influence on loyalty (Taylor, 1998; Bennet and Rundle-Thiele, 2004; Schultz, 2005; Sondoh et al., 2007). However, sports federations perceive that, despite high consumer satisfaction, the number of federated members remains stable over the years. Nowadays, they are concerned about attracting new sportsmen and sportswomen and increasing the number of members. This makes it necessary to introduce new variables in the management model of sports federations, which will have a positive influence on the loyalty of the federate and will make it possible to attract new sportsmen and sportswomen.

More loyalty studies need to be conducted to better understand this concept (Sondoh et al., 2007; Cuesta-Valiño et al., 2020) to understand that there are more variables that can be considered as the background of loyalty, in the context of karate sports federations. Therefore, one of the novelties of this study is the consideration of the consumer satisfaction variables, engagement, and meaningful as determinants of loyalty, as well as the happiness construct, which in turn is a mediator.

Research on happiness is common in the fields of psychology, education, organizational behavior, religion, tourism, and hospitality (Fu and Wang, 2020). One of the novelties of this study is the consideration of the happiness variable as an antecedent to loyalty applied to sport, specifically in the discipline of karate.

In Spanish sport, the federations function as mixed organizations, being of a public and private nature. In other words, they are private non-profit organizations, but they perform functions typical of other public services. Specifically, among the main functions of the karate federations are the promotion and encouragement of high-level and high-performance sport by Spanish karate athletes at the international level (Loranca-Valle et al., 2019).

Despite the growing interest of the general population in physical activity and sport, there has not been a proportional increase in the number of federated karate sportsmen and sportswomen in recent years (Wemmer and Koenigstorfer, 2016). For their part, for-profit sports organizations have made a very strong entry into the market and are attracting more and more sportsmen and sportswomen, to the detriment of non-profit sports organizations (Kotler, 1971; Smith and Stewart, 2010; Liu et al., 2018). Thus, the contributions of this article provide insights into the analysis of loyalty strategies that work best in the context of karate sport. In this way, the members of the Boards of Directors of the karate federations can improve their market share, gaining the loyalty of current sportsmen and sportswomen, and gaining new members.

Thus, the main objective of this research is to propose an SEM model that explains the relationships between consumer satisfaction, engagement, and meaningful and happiness as a background to loyalty. Such a model has important implications both for the contribution to literature and for the practice of loyalty marketing strategies of the federated karate athlete.

**Figure 1:**
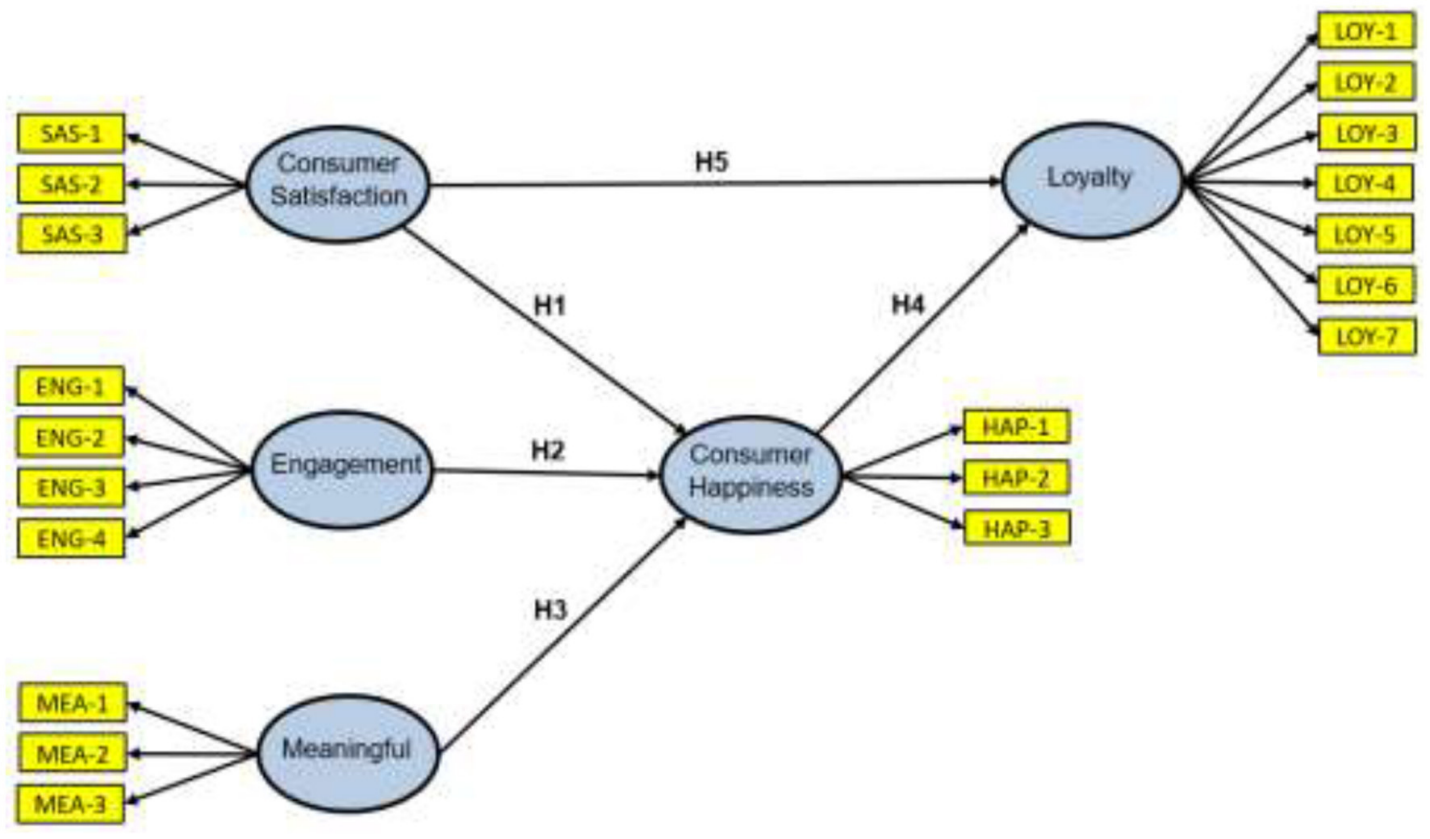
Conceptual model and hypothesis.

Figure 1 shows the model developed with each of the constructs, its dimensions, and the hypotheses to be contrasted.

Satisfaction is the most emotional antecedent to loyalty and consumer intent (Rauyruen and Miller, 2007). Satisfaction is related to the satisfaction of the consumer's needs, and when these needs are repeatedly satisfied, it is possible to lead the client to feel an emotional bond between the entity and the consumer (Stauss and Neuhaus, 1997). Therefore, satisfaction in the services provided by an entity is formed in a cumulative way with all the exchanges (Maxham and Netemeyer, 2002).

Some authors (Oliver, 1997; Szymanski and Henard, 2001) explain satisfaction as a perception of consumer response to consumption, with levels of compliance being insufficient or excessive with respect to expectations and actual implementation (Fornell, 1992; Andreassen, 2000). Other authors consider other variables such as consumption experience (Cadotte et al., 1987; Anderson et al., 1994; Sirdeshmukh et al., 2018), consumption of the ideal product or service (Tse and Wilton, 1988); equity theory (Oliver and Swan, 1989), and desires (Spreng and Olshavsky, 1993).

Perceptions derived from the experience of consumption form the basis of consumer satisfaction, and in turn, this leads to consumer happiness (Dagger and Sweeney, 2006; Anderson and Mansi, 2009; Sweeney et al., 2015; Gong and Yi, 2018). Han et al. (2019) add that satisfaction has a positive influence on consumer happiness and retention.

In the literature on consumer behavior, several authors argue that the experience of consuming a good or service translated into levels of satisfaction has a positive influence on the consumer's happiness with that consumption (Nicolao et al., 2009; Howell and Guevarra, 2013; Gilovich et al., 2015; Theodorakis et al., 2019).

Satisfaction is reflected in global feelings from the material, and experiential consumption derived from the purchase (Yoshida and James, 2010; Theodorakis et al., 2019). Thus, consumer satisfaction is reflected in the degree of a positive feeling of a client toward a service provider (Cronin et al., 2000; Deng et al., 2010).

Therefore, the following hypothesis is formulated:

*H1. Consumer satisfaction positively influences happiness*.

*Engagement* refers to feeling more secure and committed to the entity (Massimini and DelleFave, 2000; Deng et al., 2010). Engagement is also the result of immediate experience (Hom Cary, 2004; Duckworth et al., 2005). Consumers can engage when they go through active and passive experiences (Schmitt, 2012). Multiple points of contact with consumers can be created, such as events and direct interactions, which enhance the direct experience. On the other hand, the experience can be passive through traditional mass media and even make use of online media to foster brand immersion (Schmitt, 2012).

Engagement can be seen as the motivation that drives a party to trust a particular entity (Moorman et al., 1993) or as the psychological attachment to the organization (Gruen et al., 2000). The commitment implies the conviction on both sides that maintaining the relationship will be more beneficial than ending it (Theyskens et al., 1996). All definitions of engagement agree that there is a psychological component (bonding, liaison, promise, or commitment) and a motivational component (maintaining the relationship, repeat purchases, staying in the organization, Meyer and Allen, 1991; Jones et al., 2010). Consumers expressed confidence in service providers to avoid unfair and opportunistic exchanges (Gefen et al., 2003).

Commitment is a very important ingredient for the success of a business relationship (Morgan and Hunt, 1994), and it is also a key variable in marketing (Garbarino and Johnson, 1999). Commitment is closely related to reciprocity, loyalty, and the rejection of alternatives. All these variables are related to the meaning of the relationship (Gundlach et al., 1995). It is the first step in building trust in the relationship and influencing the development of social norms that regulate future exchanges (Williamson, 2007).

Many authors agree that trust is one of the basic ingredients for successful relationships (Dwyer et al., 1987; Moorman et al., 1993; Berry, 1995). Trust in an organization is based on consumer assurance of the quality and integrity of the service offered (Moorman et al., 1993; Morgan and Hunt, 1994; Garbarino and Johnson, 1999; Hennig-Thurau et al., 2001). Trust is the belief of one party that the actions of the other party will necessarily meet its needs (Anderson and Weitz, 1989). In the relationship between consumers and organizations, the psychological benefits of safety and confidence are more important than the special treatment or social benefits derived from that relationship (Gwinner et al., 1998). Trust is a participation in a process, which has been well-planned, whereas affection for the institution is spontaneous, more immediate, and less reasoned (Chaudhuri and Holbrook, 2001). Trust, as engagement, can have both affective and cognitive dimensions (Johnson and Grayson, 2005).

Some authors explain happiness as the positive psychological state derived from a good, pleasant, and satisfying experience (Jang et al., 2017; Loranca-Valle et al., 2019). Therefore, the following hypothesis is formulated:

*H2. Engagement positively influences happiness*.

There is currently a great deal of research on the economics of happiness that empirically demonstrates that money alone does not bring higher levels of subjective well-being or happiness to human beings in the era of the digital society (Powdthavee, 2010; Ravina-Ripoll et al., 2020). If this issue is examined from the perspectives of positive psychology and happiness management, it shows us, among other things, that people's happiness is conditioned by a wide range of heterogeneous factors, including health, sexual behavior, resilience, stress, or quality of life (Blanchflower and Oswald, 2004; Leite et al., 2020; Tandler et al., 2020). It is generally accepted that happiness is found, on the one hand, in the individual satisfaction that is enjoyed in the long term when our achievements and purposes are fulfilled—eudemonism—and, on the other hand, in the pleasure of carrying out rewarding activities in the short and medium term—hedonism (Huta and Waterman, 2014). Based on this philosophical and psychological adage, an important volume of scientific work emerges focused on exploring that the meaningful originates when people gravitate their individual efforts in undertaking social activities or actions that contribute to the common good and therefore to the collective happiness of the citizens (Duckworth et al., 2005; O'Donovan and Sheikh, 2014; Núñez-Barriopedro et al., 2020). In line with this research, the literature shows that meaningful, at the microindividual level, should be associated not only with negative, depressive, and unhealthy experiences, but also with positive and optimistic emotions. As is known, both elements are excellent drivers for enjoying a healthy, joyful life full of subjective well-being, i.e., good living (Li et al., 2020). This has a strongly subjective, individual, and relational component (Kok et al., 2015). If this issue is approached from the lens of religion, the predominant vectors for living a truly meaningful life will be the love of neighbor, generosity, and altruism (Nielsen, 2014). However, a review of the literature suggests the existence of limited attempts to analyze this link (meaningful–happiness) within the world of sport (Netz et al., 2008; Jang et al., 2020). Many studies have shown that meaningfully contributes holistically to good physical health and psychological well-being, especially in difficult everyday circumstances (Taylor et al., 2000; Crawford, 2006).

Along with this cognitive purpose, it cannot be overlooked that over the last decades, a significant number of academic productions have empirically shown that the happiness of human beings lies entirely in cultivating a meaningful life (Sirgy and Wu, 2009; Baumeister et al., 2013; Bartsch and Oliver, 2017). The questionnaire developed by Morgan and Farsides (2009) on the Measurement of Meaningful Living, as well as the survey designed by Steger et al. (2006) on the Meaning of Life, has undoubtedly contributed to this assumption. Both psychometric scales are characterized by assessing, among other things, whether my life is exciting or whether I am satisfied with my life project.

This growing interest leads us to propose the following hypothesis of analysis:

*H3. Meaningful positively influences happiness*.

Many studies have identified that happiness has a significant effect on loyalty (Shin, 2008; Khan and Hussain, 2013; Kim et al., 2015; Vittersø et al., 2017; Wu et al., 2017; Cuesta-Valiño et al., 2019).

The importance of making consumers happy not only involves cultivating happiness as a state but also in the favored consumption behavior that results from that state (Higgins, 1997; Fredrickson, 2001).

Also, consumers tend to repeat pleasant and happiness-fostering experiences and avoid unpleasant ones (Higgins, 1997). In this way, positive emotions have the capacity to expand the consumer's momentary thinking and facilitate the construction of lasting physical, intellectual, social, and psychological patterns of purchasing behavior (Fredrickson, 2006).

In addition, authors (Van Boven and Gilovich, 2003) explain that purchase happiness is the sum of material consumption and the experience of such consumption, which affects the consumer's choice to consume the product again and therefore has a positive influence on loyalty.

Likewise, happiness has been defined as the positive psychological state derived from a pleasant and satisfactory experience (Lyubomirsky et al., 2005a; Ahuvia, 2008). Happiness can be seen from two perspectives, one referring to a specific moment and the other to an imperishable duration in time (Ravina-Ripoll et al., 2020). The first results from a particular positive situation or experience, whereas the second is a general positive psychological state that is cumulative over time (Lyubomirsky et al., 2005b).

Loyalty comprises all those behaviors that involve the consumption of a good or service of the same entity repeatedly, both now and in the future (Gong and Yi, 2018; Septianto et al., 2019; Gómez-Suárez and Veloso, 2020), despite the influences of competitive marketing strategies (Septianto et al., 2019).

Loyalty consists of two concepts that combine to give the variable the greatest explanatory power. These two concepts are behavioral aspects or purchase intentions and attitudinal loyalty (Cossío-Silva et al., 2016). Behavioral loyalty has been identified as the willingness of customers to buy back the product or services and to maintain a relationship with the supplier or service provider (Jones et al., 2010). Attitudinal loyalty, however, is the level of consumer commitment to a product/brand and their promotional attitude toward the supplier of goods or services (Dick and Basu, 1994; Chaudhuri and Holbrook, 2001).

As a consequence of loyalty, there are authors who explain the resistance to change (Pritchard et al., 1999; Bansal et al., 2004; Bodet, 2012) and word-of-mouth (Parasuraman et al., 1988; Dick and Basu, 1994; Kim and Trail, 2011; Yaseen et al., 2011).

According to numerous studies, happiness and loyalty are positively related (Cuesta-Valiño et al., 2019). Customer satisfaction does not imply happiness, but in order to lead the customer to loyalty, we must seek his/her happiness (Khan and Hussain, 2013; Cuesta-Valiño et al., 2020) instead of focusing on his/her satisfaction, which is what has been pursued for the last 50 years (Easterlin, 2001).

Therefore, the following hypothesis can be made:

*H4. Happiness positively influences loyalty*.

Consumer loyalty to a brand is manifested in the fact that there is a commitment to future repurchase behavior of a product or service and is not affected by the marketing influences of competitors (Oliver, 1999). This definition emphasizes the two fundamental aspects of loyalty described in the literature relating to behavior and attitude (Oliver, 1999).

The behavioral component of loyalty is linked to the repurchase of a product or the degree of repeat purchase by an individual with respect to a brand, without analyzing the reasons why it occurs (Chaudhuri and Holbrook, 2001). However, the authors consider that knowing the cause of repurchase is essential to be able to talk about loyalty (Bloemer and Kasper, 1995).

In contrast, the attitudinal approach to loyalty refers to a certain degree of commitment to the brand (Chaudhuri and Holbrook, 2001) and advocates that, to measure true consumer loyalty, one must collect preferences or intentions for future behavior (Bloemer and Kasper, 1995). Even Reichheld (2003) has argued that it is possible for several service companies to properly assess loyalty using only one measure: the willingness to recommend. This feeling corresponds to a positive attitude toward the company (Dick and Basu, 1994; Barroso Castro and Martin Armario, 1999) generated by an internal evaluation process (Bloemer and Kasper, 1995), which is reflected in recommending the product or brand to others (Selnes, 1993) and in other cognitive aspects (Lee and Zeiss, 1980), such as tolerance to pay a higher price for the product (Martin et al., 2009) or being the brand that would be chosen first from a range of alternatives (Ostrowski et al., 1993). Researchers have also argued for the third dimension of service loyalty—the cognitive element (Bloemer and Kasper, 1995; Oliver, 1999)—where loyalty is based on a conscious assessment of brand attributes or the reward and benefits associated with sponsorship (Lee and Cunningham, 2001), leading the consumer to consider this service provider in relation to others (Dwyer et al., 1987).

It is widely accepted in the literature that loyalty is based on satisfaction, which acts as a background to it (Dick and Basu, 1994), helping to increase sales (Lewis, 2004). Thus, it is recognized that consumers with high levels of satisfaction also tend to be more loyal (Fornell, 1992; Bloemer and Kasper, 1995; Oliver, 1997; Chang and Tu, 2005; Li and Green, 2011). Therefore, a satisfied consumer is more likely to buy back a product (Selnes, 1993; Oliver, 1997; Baker and Crompton, 2000; McDougall and Levesque, 2000; Caruana, 2002; Olsen and Johnson, 2003; Yoon and Uysal, 2005; Mao, 2010) and become a prescriber of the product, engaging in positive word-of-mouth communication with other consumers (Andreassen, 2000; Homburg and Giering, 2001; Olsen and Johnson, 2003).

Therefore, the following hypothesis is formulated:

*H5. Consumer satisfaction has a positive influence on loyalty*.

## Methods

### Survey Design

The data to test these hypotheses were collected from a cross-sectional descriptive study. This research used primary data from a survey answered by a sample of members of karate federations in Spain from March 2019 to March 2020. The questionnaire analyzes the constructs of the proposed model with the different items. The primary selection of the different items of the five constructs was based on a review of the literature. Previously, the items had been carefully chosen, and before sending out the survey, preceding qualitative research was carried out through a focus group, which included three professors who are experts in psychology and consumer behavior, three managers who work in karate federations, and three members of karate federations.

As a result of this qualitative research, the final questionnaire was achieved, consisting of five constructors with a total of 20 items: three for consumer satisfaction (Maxham and Netemeyer, 2002; Deng et al., 2010), four for engagement (Meyer and Allen, 1991; Hennig-Thurau et al., 2001; Gefen et al., 2003), three for meaningful (Meyer and Allen, 1991), three for consumer happiness (Dagger and Sweeney, 2006; Sweeney et al., 2015; Gong and Yi, 2018; Han et al., 2019), and seven items for loyalty (Miller and Boster, 1988; Holmes and Rempel, 1989; Rauyruen and Miller, 2007; Fu and Wang, 2020). The scale used for these 20 items was a five-point Likert-type response format, in which respondents could value the items from 1 (“strongly disagree”) to 5 (“strongly agree”) (Table 1).

**Table 1:** Constructs, items, factor loading, reliability, and validity.

**Factor loadings**		**References**
**Consumer satisfaction (SAS)** RVM: Cronbach α: 0.84, AVE: 0.76, composite reliability: 0.90		
I think I did the right thing when I subscribed to this federation service.	0.79	Maxham and Netemeyer, 2002; Deng et al., 2010
As a whole, I am satisfied with the federation.	0.92	
I am satisfied with the overall service that my federation provided to me.	0.88	
**Engagement (ENG)** RVM: Cronbach α: 0.90, AVE: 0.77, composite reliability: 0.93		
I do feel “emotionally attached” to my federation.	0.86	Meyer and Allen, 1991; Hennig-Thurau et al., 2001; Gefen et al., 2003
I was proud to be able to participate in my federation.	0.88	
Based on my experience, I know the federation service provider cares about customers.	0.89	
Based on my experience, I know the federation service provider is honest.	0.89	
**Meaningful (MEA)** RVM: Cronbach α: 0.70, AVE: 0.63, composite reliability: 0.83		
I do feel like “part of the family” at my federation.	0.85	Meyer and Allen, 1991
I have friends who belong to my federation.	0.80	
I do feel a strong sense of belonging with my federation.	0.72	
**Consumer happiness (HAP)** RVM: Cronbach α: 0.83, AVE: 0.82, composite reliability: 0.93		
Participating in the activities of my federation makes me happy.	0.93	Dagger and Sweeney, 2006; Sweeney et al., 2015; Gong and Yi, 2018; Han et al., 2019
The members are happy when they participate in the activities of my federation.	0.92	
The fee I paid for participating in my federation activities was worth.	0.88	
**Loyalty (LOY)** RVM: Cronbach α: 0.94, AVE: 0.72, composite reliability: 0.95		
The relationship will remain intact well into the future.	0.82	Miller and Boster, 1988; Holmes and Rempel, 1989; Rauyruen and Miller, 2007; Fu and Wang, 2020
I will recommend others to use the federation service.	0.90	
Even if friends recommended another service, my preference for the federation service would not change.	0.81	
I am motivated to maintain the relationship into the future.	0.86	
Even there are other options to federate, I will still federate in my federation.	0.90	
I will continue to participate in activities of my federation.	0.81	
I will continue my activities in my federation before any other federation.	0.84	

*RVM, reliability and validity measures*.

A pretest of the questionnaire was carried out in February 2019 on a representative sample of members of karate federations. Some errors were then corrected, and all questions were validated. When the questionnaire had been refined, it was launched online through a discretionary non-probabilistic sampling by quotas, with the objective of completing the distribution of genders as similar as possible to that of the population who are members of karate federations in Spain, which have a total of 75,406 members. The questionnaire was distributed through the main social networks in March 2019 and through karate federation websites from March 2019 to March 2020. The result was that 714 members answered the survey, but 32 surveys were not filled in correctly, so the total number of valid questionnaires was 682, implying a sampling error of 3.81% (with a 95.5% confidence interval and *p* = *q* = 0.5).

### Sample Size and Composition

A descriptive statistic for the sample is presented below. The total sample size was 682 individuals who represent members of karate federations in Spain. The composition of the sample was 71% male and 28% female. By age group, 19% are younger than 16 years, 27% are 16–29 years old, 20% are 30–44 years old, 32% are 45–64 years old, and 2% are older than 64 years. By years of membership, 3% have <1 year, 16% have 1–5 years, 23% have 6–10 years, 25% have 11–20 years, and 32% have more than 20 years. And finally, from the point of view of Black Belt grade, the most numerous groups are members with Black Belt 1st and 2nd Dan (42%), members with less than Black Belt (29%), and members with Black Belt 3rd and 4th Dan (21%), and only 8% are members with the highest Black Belt grades (Table 2).

**Table 2:** Sample composition.

**Gender**	**Total of 682**	**%**
Male	484	71.0
Female	194	28.4
Non-responded	4	0.6
**Age (y)**	**Total of 682**	**%**
<16	130	19.1
16–29	181	26.5
30–44	135	19.8
45–64	218	32.0
More than 64	16	2.3
Non-responded	2	0.3
**Years of membership**	**Total of 682**	**%**
<1	22	3.2
1–5	109	16.0
6–10	158	23.2
11–20	172	25.2
More than 20	219	32.1
Non-responded	2	0.3
**Black Belt grade**	**Total of 682**	**%**
Less than Black Belt	195	28.6
Black Belt 1st and 2nd Dan	283	41.5
Black Belt 3rd and 4th Dan	146	21.4
Black Belt 5th and 6th Dan	38	5.6
More than Black Belt 7th Dan	19	2.8
Non-responded	1	0.1

## Results

### Measurement Model: Reliability and Validity

Partial least squares structural equation modeling (PLS-SEM) is an opportunity to advance the development and testing of theory in sport organization management. SEM has become a mainstream method in many fields of business research, and PLS-SEM provides a flexible method in terms of data requirements, model complexity, and relationship specification. PLS-SEM does not require normally distributed data (Hair et al., 2017) and is therefore the more appropriate method of SEM for many social science studies, where data are often non-normally distributed. Also, as the primary purpose in theory development is to find relationships, their directions, and strengths, as well as observable measures, PLS-SEM is appropriate.

Analyzing the proposed model, there are five different latent constructs, and each of the scales consists reflective items. The main reason that this option was selected can be held on that the effects when items are removed do not affect the content validity and the items are correlated. In the following paragraphs, it will be assessed factor loading, reliability, discriminant validity assessment, and the other measures included in the study to determine the model fit. In order to assess factor loading or indicator reliability, it is examined how each item relates to the latent constructs. Then, the outer loadings of the reflective constructs are well above the threshold value of 0.707, from which it is possible to obtain the indicator reliability. In this study, all of the 20 items reach this level of acceptable reliability because their loadings exceed 0.72 and load more highly on their own construct than on others. These results provide strong support for the reliability of the reflective measures (Table 1).

In order to measure reliability, Cronbach α is commonly used, but it has been criticized for its lower bound value, which underestimates the true reliability (Peterson and Kim, 2013). In Smart PLS 3.3.2 (Ringle et al., 2015), composite reliability (CR) is available to measure reliability because some items will be more important for your construct than others, which implies different outer loadings on the construct. These different outer loadings are taken into account when you determine the CR of your construct. Nunnally and Bernstein (1994) suggest 0.70 as a benchmark for a Cronbach α reasonable reliability, and the CR is suggested 0.80 as “stricter” reliability applicable in basic research. In this case, all constructs exceed the limit values recommended for both measures.

The discriminant validity assessment has the goal to ensure that a reflective construct has the strongest relationships with its own indicators (Hair et al., 2017). Discriminant validity assessment has become a generally accepted prerequisite for analyzing relationships between latent variables (Hair et al., 2017). Traditionally, the Fornell and Larcker (1981) criterion is used, but Henseler et al. (2014b) propose an alternative approach because the heterotrait–monotrait ratio of correlations (HTMT) detect the lack of discriminant validity in common research situations. If the HTMT value is below 0.90, discriminant validity has been established between two reflective constructs. All the HTMT coefficients in the study have a value below 0.9, and the square root of AVE is greater than the correlation between the constructs (Fornell and Larcker, 1981). This last result is referred to the Fornell–Larcker criterion, suggesting that each construct relates more strongly to its own measures than to measures of other constructs.

### Structural Model: Goodness-of-Fit Statistics

Absolute fit indices indicate how well a model fits the sample data (McDonald and Ho, 2002). Standardized root mean square residual (SRMR) was introduced by Henseler et al. (2014a) as a goodness-of-fit measure for PLS-SEM. Standardized SRMR is defined as the average magnitude of the discrepancies between observed and expected correlations as an absolute measure of (model) fit criterion. Hu and Bentler (1998) considered that a value <0.10 is a good fit, but a value <0.08 (in a more conservative version) is more recommended. In this model, SRMR is 0.055, suggesting a good fitting model. A large amount of variance is also explained in consumer happiness and loyalty, with *R*^2^ values of 0.77 and 0.78, respectively. The Stone–Geisser (Q2) results for the same variables are 0.63 and 0.56, respectively, where values larger than zero indicate a good model's predictive relevance. Predictive validity is included as the last step of confirmatory composite analysis (CCA), which is a systematic methodological process for confirming measurement models in PLS-SEM. The statistical objective of CCA and confirmatory factor analysis (CFA) is confirmation of measurement theory. To achieve measurement confirmation objectives in developing or adapting multi-item measures, researchers could use either CFA or CCA (Hair et al., 2020). These steps with reflective measurement models are significant loadings, indicator reliability, CR, AVE, discriminant validity, nomological validity, and predictive validity. All these elements have been presented previously. Also, the consistent PLS algorithm that performs a correction of reflective constructs' correlations can be used to ensure that the results were consistent with a factor model. The results are very similar, and it was not necessary to apply this algorithm.

### Results of SEM

**Figure 2:**
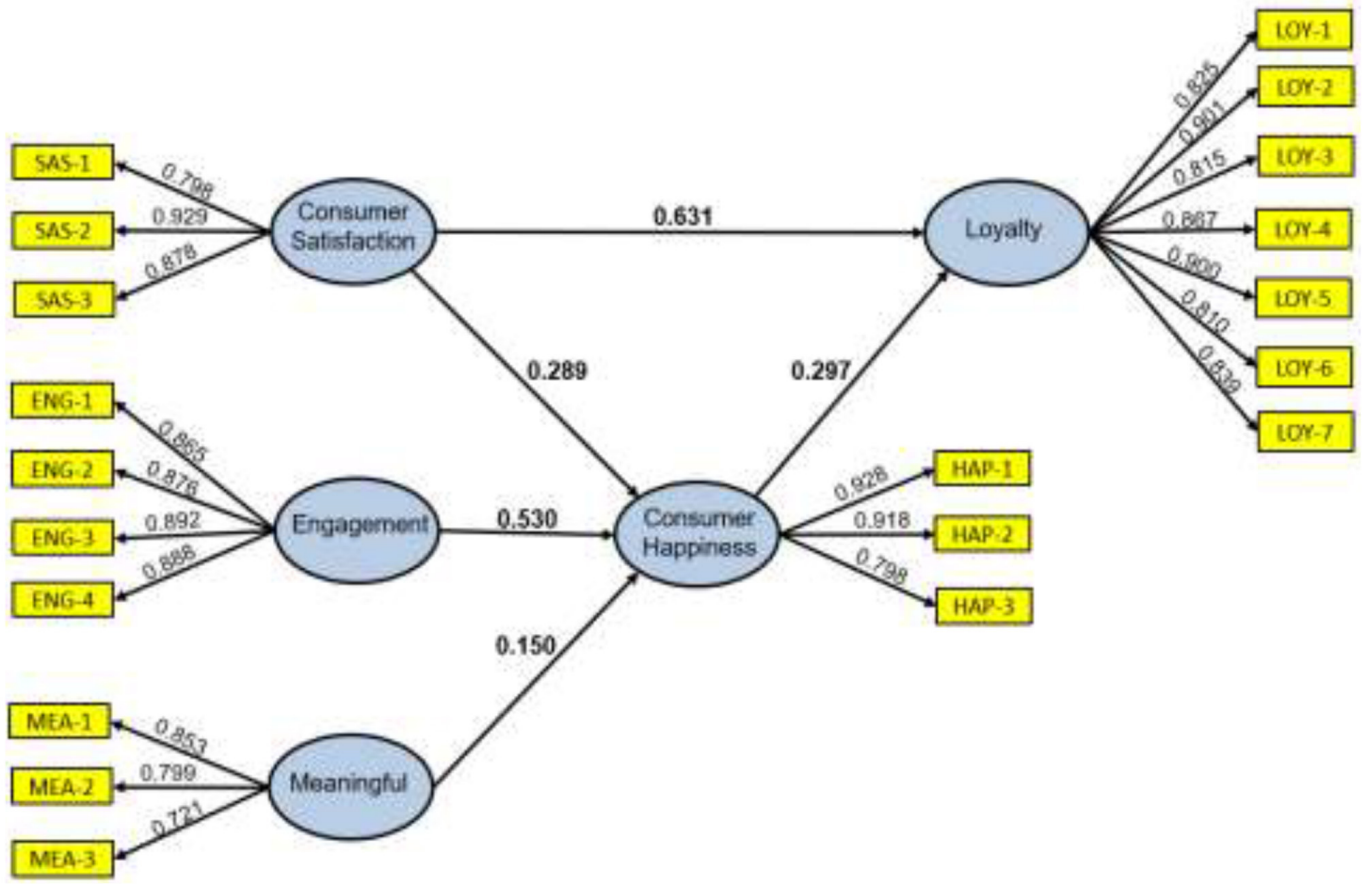
Conceptual model results.

The conceptual model results (Figure 2) show how consumer happiness is related to each of its antecedents. With a coefficient of 0.53, the results suggest that engagement influences consumer happiness in an important and positive way. This situation is followed by consumer satisfaction and meaningful antecedents that are also influencing positively but in a less relevant way. Even so, it can be said that meaningful has a weak influence (with value coefficients of 0.29 and 0.15, respectively). Therefore, the hypotheses H1, H2, and H3 are not rejected (Table 3).

**Table 3:** Summary of hypothesis verification.

**Hypothesis**	**Content**	**Verification**
H1	Consumer satisfaction has a positive influence on happiness.	Supported
H2	The engagement has a positive influence on happiness.	Supported
H3	Meaningful has a positive influence on happiness.	Supported
H4	Happiness has a positive influence on loyalty.	Supported
H5	Consumer satisfaction has a positive influence on loyalty.	Supported

For the hypothesis attempting to discover the relationship between consumer happiness and loyalty, it is very clear that the relationship is relevant and positive (0.30). The relation between consumer satisfaction and loyalty is strong and positive, with a high coefficient (0.63). Then, H4 and H5 are not rejected.

Finally, it is relevant to analyze the results of the total effects on consumer happiness and loyalty. Before, the direct effects were commented on in the hypothesis, but it is also noteworthy to know how indirect effects are influencing the total effects of engagement and meaningful on loyalty (Table 4).

**Table 4:** Total effects.

	**Consumer happiness**	**Loyalty**
Consumer satisfaction	0.289^*^	0.717^*^
Engagement	0.530^*^	0.158^*^
Consumer happiness		0.297^*^
Meaningful	0.150^*^	0.044^*^

* *Significant path coefficients (at p < 0.01)*.

## Discussion

The present study arises with the purpose of deepening the knowledge about the variables that can positively influence loyalty and specifically how happiness can generate loyalty considering each of its dimensions (Aksoy et al., 2015; Zhong and Moon, 2020) in addition to the satisfaction. For this purpose, a structural equation model (SEM) has been developed, composed of exogenous variables of positive values that aim to explain the happiness dimensionality inferentially, from the perception of consumer satisfaction, engagement, and meaningfulness. The literature shows that these three parameters are significantly associated with people's happiness, understood as a continuous state of pleasure and significance (Ltifi and Gharbi, 2015; Lera-López et al., 2020; Oishi et al., 2020). The empirical results achieved by this research are in line with our theoretical model, despite the fact that there are currently few economic and multidisciplinary studies that analyze the subject matter of our work in the field of sport and particularly in the area of karate (Loranca-Valle et al., 2019).

Reichheld (2003) and Jones et al. (2010) warn that without an understanding of the nature and extent of loyalty, service organizations may be measuring misguided elements of consumer behavior or attitudes when designing loyalty programs. While it is true that the consumer may purchase the same product by chance, lack of alternatives (Chaudhuri and Holbrook, 2001), convenience (Bloemer and Kasper, 1995), or price does not lead to true loyalty. Therefore, the contributions of this work on the dimensions that truly influence loyalty are Happiness, Satisfaction, Engagement, understood as trust, meaningful, or the pleasant life of the consumer to want to maintain the relationship or purchase the same brand and the meaning is fundamental for an efficient allocation of resources in consumer loyalty programmes.

Likewise, one of the findings of this study is that the happiness variable plays a very important role in the loyalty parameter of sportsmen and sportswomen (Gladden and Funk, 2001) in a study environment that has not been explored until now. This suggests that loyalty to this sport is associated with the happiness of its practitioners whenever high levels of engagement are experienced (0.526), which should be accompanied by moderate levels of meaningful (0.150) and consumer satisfaction (0.293). This interpretation is also consistent with previous studies, such as the work of Baena-Arroyo et al. (2020), who empirically demonstrate that the happiness or satisfaction of people who practice sports is strongly conditioned by the quality of the services offered by sports organizations. In this sense, the study by Mirehie and Gibson (2020) should also be noted. This study shows that women who practice skiing and snowboarding enjoy a high level of psychosocial well-being, as the practice of this leisure activity provides them with high doses of positive emotions, commitment, and meaning.

In line with this, our quantitative results reveal that happiness needs to be given greater consideration in its association with loyalty in the context of sport. This gap constitutes a great opportunity to put on the academic agenda that happiness is understood as a strategic differential factor that motivates people to be loyal to practice a sport assiduously under the lens of consumer satisfaction, engagement, and meaningful. This is extremely important as the continuous practice of physical exercise contributes to holistically improve people's health and psychological well-being, factors that cushion stress, depression, vascular diseases, etc. (Ronkainen et al., 2020).

Regarding the practical implications of our work, on the one hand, public institutions can encourage the regular practice of sport to citizens through happiness, as emotional health, self-esteem, cognitive performance, commitment, confidence, socialization, etc. (Jiménez-Marín et al., 2020). On the other hand, it is recommended that sports federations implement Certification Happiness Management in the future as an instrument that certifies that, within these organizations, the collective well-being of all their human capital and stakeholders is cultivated under the guiding principles of the collaborative economy, philosophy, corporate social responsibility, and positive psychology. In this way, sports companies can carry out strategic actions aimed at building positive emotions and satisfactory experiences that help to engage their potential consumers. In light of the above, we believe that a very promising scenario is opening up in the post–COVID-19 era, as well as many opportunities for entities that actively develop this type of culture, marketing, and business management based on the virtuous circle of corporate happiness (Ravina-Ripoll et al., 2020).

Among the limitations of this study is that a transversal study has been designed so that in future research, longitudinal studies can be carried out based on panel data to evaluate the impact of the variables evaluated in the model presented in this work. The geographical area studied was Spain, where karate members from all the Autonomous Communities formed part of the sample for this study. However, in future work, the sample could be extended to other countries. Another limitation is the application of the study to the discipline of karate so that, in future research work, the proposed model can be applied to other sporting disciplines.

Finally, future research on happiness management in the sports context should be focused on exploring whether the individual happiness provided by the daily practice of sport is significantly correlated with other psychological dimensions or with quantitative indicators of subjective well-being. In this way, we will be able to obtain, on the one hand, empirical evidence of new constructs associated with happiness in the environment of sports participation, both an active and passive nature. On the other hand, we can analyze, in a multidisciplinary and cross-cultural way, how daily physical exercise is linked to a meaningful and healthy life, etc., and therefore full of happiness in the broad sense of the word. Hence, the interest in promoting new works can empirically confirm the subject of this research from our structural equation model, bearing in mind that the practice of sport is an important determinant of people's happiness, which is also affected by other socioeconomic and psychological elements.

## Conclusion

This study shows how the consumer happiness of the karate federation members could be a good strategy for developing loyalty in this non-profit sports organization. In an overall model, consumer satisfaction is still a well-established variable for influencing loyalty, but consumer happiness plays a mediator role with consumer satisfaction, and it is able to increase loyal members if federations principally work on engagement activities. It would therefore be interesting for karate sports federations to use this information when developing new strategies for improving loyalty.

## Data Availability Statement

The raw data supporting the conclusions of this article will be made available by the authors, without undue reservation.

## Author Contributions

EN-B, PC-V, PG-R, and RR-R: conceptualization, methodology, validation, formal analysis, investigation, resources, data curation, writing—original draft preparation, writing—review and editing, and supervision. EN-B and PG-R: software. PC-V: funding acquisition. All authors have read and agreed to the published version of the manuscript.

## Conflict of Interest

The authors declare that the research was conducted in the absence of any commercial or financial relationships that could be construed as a potential conflict of interest.
